# Dynamics of the Epigenome, Microbiome, and Metabolome in Relation to Early Adiposity in the Maternal–Infant Axis: Protocol for a Prospective, Observational Pilot Study in the Spanish NEMO Cohort

**DOI:** 10.3390/jcm14196694

**Published:** 2025-09-23

**Authors:** María Suárez-Cortés, Almudena Juan-Pérez, Alonso Molina-Rodríguez, Julia Araújo de Castro, María Ángeles Castaño-Molina, Virginia Esperanza Fernández-Ruiz, Almudena Jiménez-Méndez, Paula Martínez Pérez-Munar, Sara Rico-Chazarra, Bruno Ramos-Molina, Manuel Sánchez-Solís, José Eliseo Blanco-Carnero, Antonio José Ruiz-Alcaraz, María Ángeles Núñez-Sánchez

**Affiliations:** 1 Obesity, Diabetes and Metabolism Group, Biomedical Research Institute of Murcia (IMIB) Pascual Parrilla, 30120 Murcia, Spain; maria.suarez@um.es (M.S.-C.); mangeles.castano2@carm.es (M.Á.C.-M.); virginiaesperanza.fernandez@um.es (V.E.F.-R.); paula.martinezp1@um.es (P.M.P.-M.); sara.ricoc@um.es (S.R.-C.); bruno.ramos@imib.es (B.R.-M.); 2 Department of Nursing, Faculty of Nursing, University of Murcia, 30120 Murcia, Spain; alonso.molina@um.es; 3 Murcia Health Service (Servicio Murciano de Salud), 30100 Murcia, Spain; almudena.juan2@carm.es (A.J.-P.); ajm40r@ad.sms.carm.es (A.J.-M.); jeblancoc@gmail.com (J.E.B.-C.); 4 Gynecology and Obstetrics Service, Virgen de la Arrixaca University Hospital, 30120 Murcia, Spain; 5 Pediatric Service, Virgen de la Arrixaca University Hospital, 30120 Murcia, Spain; araujo_jul@gva.es; 6 Department of Endocrinology and Nutrition, Virgen de la Arrixaca University Hospital, 30120 Murcia, Spain; 7 Pediatric Respiratory and Cystic Fibrosis Unit, Virgen de la Arrixaca University Children’s Hospital, 30120 Murcia, Spain; msolis@um.es; 8 Pediatric Research Group, Biomedical Research Institute of Murcia (IMIB) Pascual Parrilla, 30120 Murcia, Spain; 9 Gynecology, Reproduction, and Maternal-Fetal Medicine, Biomedical Research Institute of Murcia (IMIB) Pascual Parrilla, 30120 Murcia, Spain; 10 Department of Biochemistry and Molecular Biology B and Immunology, University of Murcia, 30120 Murcia, Spain; 11 Innate Immunity in Health and Disease, Biomedical Research Institute of Murcia (IMIB) Pascual Parrilla, 30120 Murcia, Spain

**Keywords:** childhood obesity, maternal-infant axis, non-invasive biomarkers, microbiome, metabolome, epigenetic changes

## Abstract

**Background:** Childhood obesity has reached epidemic levels in developed countries and is an emerging concern in developing regions. Children with excess weight are more likely to maintain this condition over time into adulthood and face a higher risk of developing metabolic disorders such as type 2 diabetes, hypertension, metabolic dysfunction-associated liver disease, and dyslipidemia. Early identification of obesity risk is, therefore, a key public health challenge. **Methods:** This is an observational, prospective, single-center cohort pilot study in 66 mother–infant dyads recruited at the Gynecology and Obstetrics Service of the Virgen de la Arrixaca University Hospital (Murcia, Spain). The primary objective is to identify early-life, non-invasive biomarkers associated with increased adiposity by integrating multi-omics approaches and analyzing maternal–infant interactions. Pregnant women will be enrolled during the third trimester and will undergo a baseline visit at 38 weeks of gestation for clinical and anthropometric assessment. Buccal swabs and fecal samples will be collected at baseline and in the peripartum period for epigenetic (DNA methylation), metagenomic, and metabolomic analyses. Infants will be evaluated at birth and followed at 6 months, 1 year, 2 years, and 3 years. Each visit will include detailed anthropometric measurements, along with collection of buccal swabs and fecal samples for multi-omics profiling. **Conclusions:** This multidisciplinary study aims to assess how maternal factors influence infant epigenetic and microbial patterns, and their relation to adiposity development. Early identification of such biomarkers may guide personalized prevention strategies and reduce the long-term burden of obesity-related comorbidities.

## 1. Introduction

Childhood obesity has reached epidemic levels in developed countries and is becoming a major cause for concern in the developing world. According to the World Health Organization (WHO), in 2020, over 39 million children under 5 years of age were affected by overweight or obesity [1,2]. In Europe, nowadays, obesity represents a major public health challenge, with a significant socio-economic burden associated with its management [3]. In fact, children with excess weight are more likely to maintain this condition over time and are at increased risk for long-term metabolic diseases, such as insulin resistance, type 2 diabetes mellitus, hypertension, metabolic dysfunction-associated liver disease, obstructive sleep apnea, and dyslipidemia [4,5,6,7,8]. The causes of childhood obesity are complex and multifactorial, including the interaction between genetics, environmental and developmental factors, and gut microbiota [9,10,11,12]. In addition, the risk of developing long-term diseases such as obesity is significantly higher in children born to both women with obesity at the beginning of pregnancy and those with excessive weight gain during gestation [13,14,15].

In recent years, the number of studies focused on identifying early-life predictors of obesity risk has increased. Currently, the most widely accepted predictors include child size, growth velocity (mainly in the first months of life), and neonatal adiposity. Evidence indicates that high neonatal adiposity, greater body size, and rapid weight or fat gain during the early postnatal period are all associated with an increased risk of long-term obesity [16,17,18], as well as a higher likelihood of developing other metabolic disorders [19,20], hypertension [21], and asthma [22,23].

Although the genetic factors involved in the development of childhood obesity are only partially understood, several studies have associated different epigenetic changes during the first months of life and the risk of long-term obesity. Within these epigenetic changes, the analysis of modifications in DNA methylation is the most studied in relation to increased adiposity [24]. In the case of adiposity in newborns, these epigenetic changes are mainly influenced by maternal environmental factors, such as obesity at the time of conception or lifestyle during pregnancy [25,26,27]. For example, it has been described that the methylation of the cg11531579 (CHFR) site is related to rapid weight gain at early ages and the development of long-term obesity [24].

On the other hand, in recent years there has been increasing evidence about the role of the gut microbiota and its derived metabolites as epigenetic regulators [28]. Such epigenetic modifications can lead to profound reprogramming in the host genome, thus altering the transcriptional machinery of cells and sensitizing them to different stimuli that affect both physiological processes [29] and the development of metabolic diseases, including obesity [30]. The establishment of the gut microbiota is a complex process that occurs in a stepwise manner and involves a dynamic evolution in abundance and diversity [31]. Although the first 2 years of life are essential for the establishment of the microbiota, there are two critical moments that affect intestinal microbial colonization, which are the first month of life, and at weaning after the introduction of new foods [32,33,34,35,36]. These represent the periods of maximum immune and homeostatic reorganization and have a great influence on the growth and the development of the individual [37]. In fact, several studies have linked alterations during the first years of live in the gut microbiota ant its derived metabolites such as short-chain fatty acids (SCFA), branched-chain amino acids (BCAA), and bile acids, to the development of various diseases, including obesity [38,39,40,41,42]. However, the molecular mechanisms by which the gut microbiota and its derived metabolites directly or indirectly influence epigenetic changes involved in the development of obesity during this critical early-life period remain largely unknown.

In this study, we aim to identify potential non-invasive biomarkers related to adiposity increase during early life by integrating various omics approaches. To achieve this, non-invasive sample collection methods will be used, which is particularly relevant when referring to early infancy, as it may provide an effective tool for the prediction and/or prevention of the development of childhood obesity and associated metabolic diseases.

## 2. Materials and Methods

### 2.1. Study Design

A single-center, observational, prospective cohort pilot study will be conducted with maternal–infant dyads at the Gynecology and Obstetrics Service and the Pediatrics Service of the Virgen de la Arrixaca University Hospital (HCUVA) in Murcia, Spain. Healthy pregnant women attending the third-trimester ultrasound appointment (32–36 weeks) at the Gynecology and Obstetrics Service, and who meet the inclusion criteria, will be recruited. Their child will be followed for the first 3 years of life.

### 2.2. Study Objectives

The main objective of this study is to identify potential non-invasive biomarkers related to adiposity during early life by integrating various omics approaches.

To achieve this, three specific aims are defined:

- To determine DNA methylation patterns, gut microbiota composition, and metabolomic profiles at different early-life stages to identify potential biomarkers related to increased adiposity.
- To establish the relationship between changes in epigenetic, microbial, and metabolomic profiles during the first years of life.
- To evaluate the influence of maternal pregestational epigenetic, microbial, and metabolomic patterns on changes in neonatal adiposity and to establish links with corresponding neonatal patterns.

### 2.3. Study Settings

Volunteers will be recruited at the Gynecology and Obstetrics Service of the HCUVA. Each pregnant volunteer will be assigned a consecutive numerical code upon inclusion in the study. Likewise, the corresponding offspring samples will be assigned the same numerical code, followed by a letter.

### 2.4. Eligibility Criteria

#### 2.4.1. Inclusion Criteria

Pregnant women meeting the following criteria will be eligible:

- Age between 18 and 45 years of age.
- Within the third trimester of gestation.

#### 2.4.2. Exclusion Criteria

Participants (mothers and/or children) who fulfill any of the following criteria will be excluded from the study:

- Toxic habits during pregnancy.
- Volunteers who suffer from autoimmune disease.
- Volunteers who suffer or have suffered from cancer.
- Severe malformation of the fetus.
- Multiple pregnancy.
- Pregestational diabetes.
- Preeclampsia.
- Altered mental state that prevents understanding of the informed consent process and/or completion of the study.
- Birthweight below the third percentile.
- Children requiring hospitalization by pediatrics immediately after birth.
- Antibiotic consumption.
- Consumption of probiotics at least within the month prior to sample collection.
- Any condition that, in the judgement of the investigators, could interfere with the study objectives or participant safety.

### 2.5. Consent

Informed consent will be obtained from all women who wish to participate in the study by the medical specialist or research nurse at the Gynecology and Obstetrics Service. A member of the research team will provide volunteers with detailed information about the objectives and the methodology of the study and will give them a written Information Sheet (Supplementary Material). Similarly, both parents will be informed of the objectives and methodology of the study for the inclusion of the child and the informed consent form must be signed by both parents (when applicable). The Declaration of Helsinki will be followed at all stages of the study.

### 2.6. Withdrawal

The subjects participating in the study may withdraw or revoke consent at any time without giving explanation and without liability or prejudice to them. Individuals who withdraw from the study will not be further followed up and will not be replaced by new participants. The researchers/clinicians may withdraw a subject from the study if they consider that the subject can no longer comply with all the requirements of the study or if any of the procedures are considered as potentially harmful to the subject or the child. Data already collected on withdrawn subjects will be retained and used for analysis, but no new data will be collected after withdrawal. Participants (mothers and children) who meet any of these criteria will be withdrawn from the study:

- Presence of adverse events that at the discretion of the investigator implies the withdrawal of the subject.
- Deviation from the protocol that affects the interpretation of the results of the study and its scientific validity.
- Medical decision.
- Resignation of the individual to continue in the study.
- Loss of follow-up.

### 2.7. Safety Assessment During Childbirth

Both vaginal and cesarean deliveries will be supervised and assisted by experienced professionals from the Gynecology and Obstetrics Service of the HCUVA following the standard protocol of the service, and without any influence of the study participation on clinical decision-making. Nevertheless, the safety of the volunteers and their offspring will be carefully monitored at all times, both during their admission to the hospital and their subsequent discharge, as well as during the various post-delivery follow-up visits to the hospital. This procedure will be routinely carried out by the staff of both the Gynecology and Obstetrics Service and the Pediatrics Service of the HCUVA.

### 2.8. Research Ethics Approval

This study will be carried out in the HCUVA in Murcia, Spain, in accordance with the current Spanish legislation that regulates biomedical research projects. The study has already obtained written authorization from the Research Ethics Committee (internal code 2023-2-13-HCUVA). All procedures comply with the ethical principles of Good Clinical Practice (GCP), the Declaration of Helsinki and all applicable regulations and the current legislation governing biomedical research, as well as with the current national and European data protection regulations, including General Data Protection Regulation (GDPR, EU 2016/679).

### 2.9. Protocol Amendments

Except in the case of emergency situations, no changes or deviations from the study protocol will be permitted without prior documented approval by the Research Ethics Committee. The committee must be informed of any proposed modification and must provide written approval for any changes or deviations that could adversely affect the rights of the volunteer or the integrity of the research. This requirement does not apply to changes intended solely to minimize inconvenience or prevent risks to participants, or to modifications related to the administrative aspects of the study.

### 2.10. Outcomes

The primary outcome of this study is to determine whether the dynamics of changes in the epigenome, metabolome, and microbiome in mother–infant dyads are associated with increased adiposity during the first years of life, and whether these changes can serve as early biomarkers for future metabolic risk. Secondary outcomes include the detailed characterization of the epigenetic profile using buccal swab samples, analysis of microbiota composition, diversity, and richness from stool samples, and targeted metabolomic profiling of bile acids, SCFAs and BCAAs in stool samples.

### 2.11. Participant Timeline

**Figure 1:**
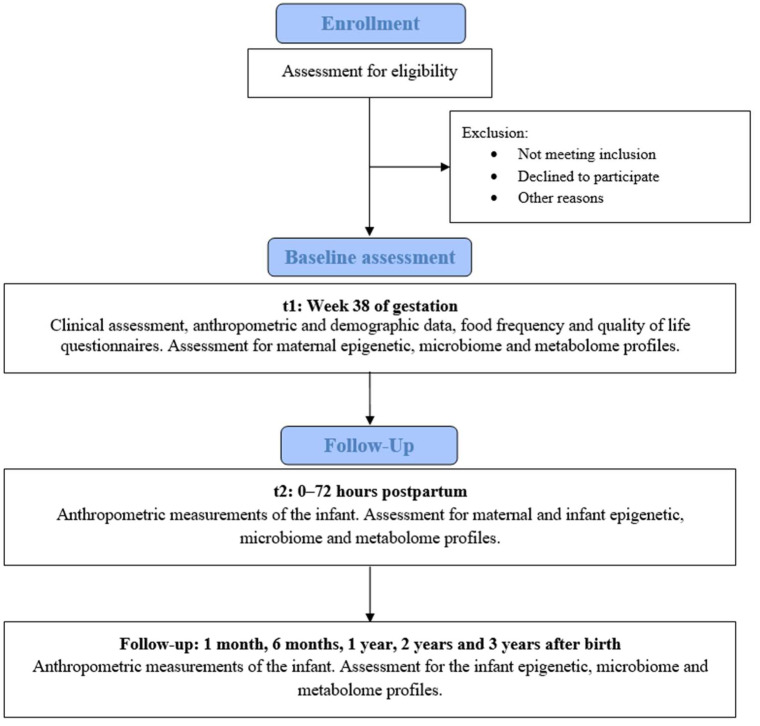
Flowchart of participant inclusion and follow-up.

Eligible volunteers who agree to participate will be enrolled and evaluated by a gynecologist. The mother will be assessed in week 38 of pregnancy and on the day of delivery, and the child will be evaluated at birth, and followed up at 1 month, 6 months, 1 year, 2 years, and 3 years after birth (Figure 1). A detailed assessment schedule is provided in Table 1.

### 2.12. Sample Size

This is a pilot observational study aiming to explore potential associations between dynamic changes in the epigenome, metabolome, and microbiome of mother–infant dyads and subsequent increases in infant adiposity. Based on different previous studies on the association of both the microbiome and epigenetic changes with adiposity [24,43,44], to reach a significance level of 0.05 and a statistical power (β) of 0.8, we established an *n* = 51 of mother–child pairs. A loss rate of around 30% is expected; 66 mother–child pairs will be included in the study. This pilot study will provide preliminary data to inform the design and sample size calculation for a larger study, which will be powered to detect statistically significant associations.

### 2.13. Data and Sample Collection and Analysis of Variables

#### 2.13.1. Data Collection

**Figure 2:**
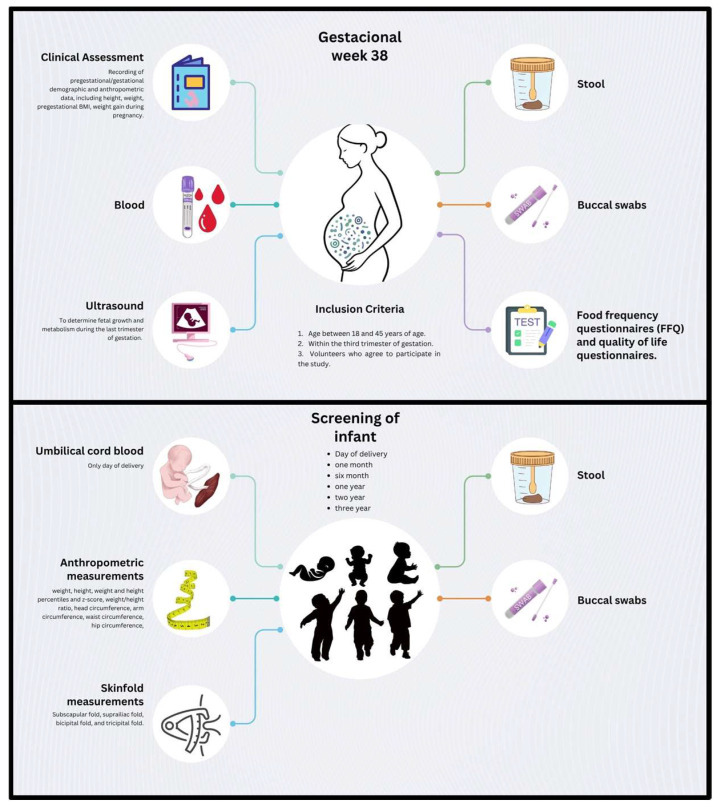
Overview of the data and sample collection in the study protocol. Schematic representation of the data and biological samples to be collected throughout the study. Maternal blood, stool, and buccal swab samples will be collected at week 38 of gestation (t = 1). During this visit, an ultrasound, clinical assessment, and food frequency questionnaires will also be conducted. Offspring stool and buccal swab samples, along with anthropometric measurements, will be collected at birth (t = 2), 1 month (t = 3), 6 months (t = 4), 1 year (t = 5), 2 years (t = 6), and 3 years (t = 7) of age.

Data and biological samples from participating mother–infant dyads will be collected at multiple time points throughout the study (Figure 2). Maternal data and samples will be obtained during the third trimester of pregnancy (gestational week 36) and on the day of delivery. Offspring data and samples will be collected at birth (within 0–48 h postpartum) and during follow-up visits at 1 month, 6 months, 1 year, 2 years, and 3 years of age.

At the maternal third-trimester visit, a complete clinical evaluation will be conducted. This will include the collection of sociodemographic information, obstetric history, and anthropometric measurements (height, weight, pregestational BMI, and gestational weight gain). Nutritional status will be assessed through a validated Food Frequency Questionnaire (FFQ) [45,46], maternal well-being will be evaluated using a standardized quality of life questionnaire, and eating behavior and weight-related attitudes will be evaluated with the Pregnancy Eating Attitudes-Questionnaire (PEA-Q) [47]. Additionally, all participants will undergo an ultrasound examination between 36 + 0 and 37 + 6 weeks of gestation, including fetal biometry for growth assessment, Doppler evaluation of the fetal and placental compartments, and measurement of fetal metabolic and adiposity indicators.

For the infant participants, detailed anthropometric data will be recorded at each time point. Measurements will include weight, height/length, corresponding percentiles and z-scores, weight-for-height ratio, head circumference, mid-upper arm circumference, waist circumference, hip circumference, and skinfold thickness at the subscapular, suprailiac, bicipital, and tricipital sites. These measurements will be conducted by trained personnel using standardized procedures. Total fat mass will be estimated using the skinfold data, measured with a Holtain skinfold caliper (Holtain Ltd., Bryberian, UK), and calculated using validated equations [48].

#### 2.13.2. Sample Collection and Storage

Biological samples collected from mothers will include saliva, blood, and stool. From infants, saliva and stool samples will be collected at scheduled time points (Figure 2).

All samples will be collected using sterile techniques. Stool samples will be transferred into sterile containers, kept at 4 °C during short-term handling, and subsequently stored at −80 °C. Buccal swabs will be collected at delivery and follow-up visits using the Buccal Swab Collection & Stabilization Kit (Canvax Biotech, Valladolid, Spain) and stored at −80 °C. Blood samples will be collected into EDTA tubes, processed, and immediately frozen at −80 °C.

All biospecimens will be stored at the Biomedical Research Institute of Murcia (IMIB) Pascual Parrilla Biobank, which is registered in the Spanish Biobank Registry of the Carlos III Health Institute (reference number PT17/0015/0038), ensuring compliance with national biobanking standards and ethical guidelines.

### 2.14. Epigenetic Changes

DNA methylation levels at CpG sites of target genes will be performed on DNA obtained buccal swab samples. Buccal swabs will be collected using the Buccal Swab Collection & Stabilization Kit (Canvax, Valladolid, Spain) and DNA will be extracted using the HigherPurity™ Buccal Swab DNA Extraction Kit (Canvax) following manufacturer’s instructions. DNA (50 ng) will be subjected to bisulfite treatment using the EZ DNA methylation kit (Zymo Research, Orange, CA, USA), according to the manufacturer’s instructions. The conversion cycling conditions will be as follows: 95 °C for 30 s and 40 °C for 60 min, repeated for 16 cycles. DNA will be then eluted in 10 µL of elution buffer. A total of 25 ng of bisulfite-treated DNA will be used for PCR amplification in a final reaction volume of 25 µL, using the PyroMark PCR kit (Qiagen, Aarhus, Denmark) and gene-specific oligonucleotides. PCR products (2 µL) will be checked on 2% agarose gel prepared in Tris-acetate-EDTA (TAE) buffer to verify the presence of a single band of the expected size for each amplicon. Pyrosequencing will be carried out in duplicate using the PyroMark Q24 system (Qiagen) and PyroMark Gold Q24 reagents (Qiagen) at the Genomic Platform of the IMIB, targeting the CpG-rich regions within the amplified sequences. Pyrosequencing run files (*.pyrorun) will be automatically analyzed using the PyroMark Q24 Software (v.2.0.8), which calculates the methylation percentage at each CpG position and provides a quality assessment of each site.

### 2.15. Analysis of Microbiota Composition

Analysis of microbiota composition will be performed on stool samples from the mother and the infant collected at different time points. Microbiota profiling will be conducted using 16S rRNA gene sequencing. Bacterial DNA will be extracted using the NZY Microbial gDNA Isolation Kit (NZYtech), according to the manufacturer’s protocol. DNA concentration and integrity will be assessed using the Agilent 2200 TapeStation system (Agilent Technologies, Santa Clara, CA, USA).

For library preparation, DNA samples (5 ng/µL) will be amplified using primers targeting the hypervariable regions V3-V4 regions of the bacterial 16S rRNA gene with the 16S Metagenomics Kit (Thermo Fisher, Waltham, MA, USA). Resulting amplicons will be purified using AMPure^®^ XP magnetic beads (Beckman Coulter, Pasadena, CA, USA). Libraries will be constructed with the Ion Plus Fragment Library Kit and barcoded using the Ion Xpress Barcode Adapters 1–16 Kit (Thermo Fisher). Final libraries will be purified with AMPure^®^ XP beads and quantified using fluorometric methods. Sequencing will be performed using the Illumina MiSeq system (Illumina, San Diego, CA, USA). Microbial composition, diversity, and richness metrics will be analyzed using QIIME2 software (version 2025.4), and predictive functional profiling will be conducted using the PICRUSt package (version v2.6.2).

### 2.16. Targeted Metabolomic Analysis

The analysis of bile acid profiles will be conducted on fecal samples collected at the same time points as the metagenomics samples. This will be performed by UHPLC (Infinity 1290; Agilent, Santa Clara, CA, USA) coupled to a high-resolution mass spectrometer with a quadrupole time-of-flight mass analyzer (6550 iFunnel Q-TOF LC/MS; Agilent) with an Agilent Jet Stream (AJS) electrospray (ESI), following their established protocols [49]. Raw data analysis will be performed using Profinder 10.0 software (Version B.10.0, Agilent Technologies) with a batch-targeted feature extraction method based on an internal bile acid database. Following data processing, the identified bile acids will be analyzed using Mass Hunter Qualitative 10.0 software (Version B.10.0, Agilent Technologies).

The analysis of SCFAs and BCAAs will be performed on stool samples mixed with an aqueous NaOH solution (5 mM) containing the internal standard (D3-caproic acid). The samples will then be centrifuged, and the supernatant will be derivatized. The samples will then undergo a two-step extraction process with the sequential addition of n-hexane, followed by centrifugation. The upper n-hexane layers containing the derivatives will be collected for analysis using gas chromatography-mass spectrometry (GC-MS). The quantification of SCFA, including acetic acid, propionic acid, butyric acid, isobutyric acid, methyl-butyric acid, valeric acid, isovaleric acid, caproic acid, and heptanoic acid, will be performed using an Agilent 7890B GC coupled to an Agilent 5977A mass spectrometer in selected ion monitoring (SIM) mode. The separations will be achieved using an HP-5MS capillary column (30 m × 250 µm i.d., 0.25 µm film thickness). Derivatized sample extracts will be injected in split mode with a 10:1 ratio, and the solvent delay will be set at 2.36 min. The temperature program will start at 50 °C for 2 min, increase to 70 °C at a rate of 10 °C per minute, to 85 °C at a rate of 3 °C per minute, to 110 °C at a rate of 5 °C per minute, to 290 °C at a rate of 30 °C per minute, and will then be held at 290 °C for 8 min. Helium will be used as the carrier gas at a constant flow rate of 1 mL per minute through the column. The temperatures of the front inlet, transfer line, and electron impact (EI) ion source will be set at 260 °C, 290 °C, and 230 °C, respectively, with an electron energy of −70 eV. Compound quantification will be carried out using calibration curves based on serial dilutions of analytical standard mixtures.

### 2.17. Statistical Analysis

To assess the distribution of continuous variables, the Kolmogorov–Smirnov test will be used for cross-sectional analyses and the Shapiro–Wilk test for longitudinal data. Descriptive statistics will summarize mother–infant characteristics at each time point, with continuous variables reported as mean ± standard deviation or median and interquartile range, and categorical variables as frequencies and percentages. Longitudinal analyses will be performed using mixed-effects models to evaluate changes in adiposity and related outcomes over time, accounting for within-subject correlations and adjusting for relevant covariates such as maternal BMI, mode of delivery, and type of feeding. In addition, stratified analyses will be planned for these factors in order to explore their potential modifying effect on primary outcomes. To explore the associations between omics-derived variables (including microbiota, epigenetic, and metabolomic data), correlation analyses (Pearson or Spearman, as appropriate), and multivariable regression models (linear or logistic) will be conducted. Unsupervised and supervised multivariate approaches, such as principal component analysis (PCA), partial least squares discriminant analysis (PLS-DA), and hierarchical clustering, will be used to identify patterns and variability within high-dimensional omics datasets. Data integration techniques, including canonical correlation analysis (CCA) and machine-learning methods like random forest algorithms, will be employed to uncover potential predictive biomarkers of increased adiposity. Missing data will be addressed using multiple imputation techniques or mixed models that inherently account for missingness. All statistical analyses will be conducted using SPSS version 28.0 (IBM Corp., Armonk, NY, USA) and R (version v.4.5.1, including packages such as lme4, vegan, and mixOmics).

### 2.18. Data Management

An electronic Data Collection Notebook will be created in which all variables relevant to the study will be recorded. A formulary will be developed for the collection of information in SPSS v.22.0 software (SPSS Inc., Chicago, IL, USA).

The variables to be collected in the record will be the following:

1. Identification data of the physician responsible:
  - Name.
  - Work center.
2. Administrative data of the intervention:
  c. Date of ultrasound.
  d. Date of delivery.
  e. Date of the first follow-up visit of the newborn (1 month).
  f. Date of the second follow-up visit of the newborn (6 months).
  g. Date of the third follow-up visit of the newborn (1 year).
  h. Date of the fourth follow-up visit of the newborn (2 years).
  i. Date of the fifth follow-up visit of the newborn (3 years).
3. Demographic data:
  j. Age.
  k. Ethnicity.
  l. Gender of the child.
4. Maternal antepartum data:
  m. Physical examination: pregestational and gestational anthropometric data.
  n. Quality of life questionnaire.
  o. Nutritional questionnaire.
  p. Personal and medical history, including arterial hypertension, diabetes mellitus, and cardiovascular, hepatic, respiratory or renal diseases, as well as chronic consumption of toxic substances (alcohol, tobacco), or chronic consumption of drugs (including anti-inflammatory drugs, immunosuppressants, etc.).
  q. Ultrasound data: fetal growth assessment, hemodynamic evaluation of the maternal-fetal compartment, and evaluation of fetal adiposity.
5. Maternal data after childbirth:
  r. Type of delivery.
6. Data of the neonate after delivery:
  s. Anthropometric data obtained by physical examination.
7. Follow-up data of the neonate:
  t. Anthropometric data obtained by physical examination at 1 month, 6 months, 1 year, 2 years and 3 years.
  u. Type of nutrition; breast milk, formula, or combined.
  v. Adverse events.

All documentation related to the study will remain stored at the participating center under the custody of the Principal Investigator until the end of the study. Once the study is completed, the documentation will be indexed and passed to the general archive of the research center, complying with the recommendations established with respect to the Good Clinical Practice Guidelines.

Data collected from participants that withdraw from the study will be retained following the guidelines from the Office for Human Research Protections. No financial incentives will be provided to any of the participants.

### 2.19. Study Limitations

This project involves a longitudinal observational clinical study spanning over 3 years (from 1 month before to 3 years after delivery). Given the extended follow-up period, some participants may face challenges in completing all scheduled visits. Despite the expected high dropout rate (estimated at 30%), the annual birth rate at the HCUVA exceeds 6500 deliveries, ensuring feasibility in recruiting the proposed cohort of 66 mother–infant dyads. Close monitoring by our clinical collaborators is anticipated to mitigate any loss to follow-up and promote participant compliance. Also, this is a single-center study and, therefore, the findings may not be generalizable to populations with different exposomes or genetic backgrounds. In addition, the observational nature of the study may make it difficult to establish causality among the variables determined. However, the prospective design, the well-characterized cohort, and the longitudinal data collection will enhance the reliability of temporal associations between potential biomarkers and dynamic changes over time related to obesity risk.

Although the initial 3-year follow-up might not be sufficient to evaluate long-term health outcomes, it covers critical developmental windows for adiposity programming and metabolic risk, which could offer valuable information related to the early pathophysiology of obesity. Finally, while the sample size is sufficient for exploratory biomarker discovery, it may not allow detection of small size effect. However, this study could generate valuable information that will guarantee further large-scale investigations.

## 3. Discussion

This study aims to identify early non-invasive biomarkers during the first 3 years of life that could help to predict the rise in adiposity and, therefore, an increased metabolic risk later in life. Childhood obesity has become one of the major health concerns worldwide due to its significant implications for long-term metabolic health [3]. Although Health Systems are developing strategies to manage this issue, early prediction and prevention remain challenging due to the complex and multifactorial nature of this disease [50]. In this regard, prenatal and postnatal environmental factors have been described to influence epigenetic modification that affects the metabolic state of the offspring and, therefore, to promote the development of different metabolic conditions later in life [51,52,53,54,55]. Similarly, alterations in gut microbiome colonization and its associated metabolites, such as SCFAs or bile acids, have also emerged as critical regulators of host metabolism and immune development [56,57,58,59,60]. Given their role in modulating epigenetic marks, the study of microbiome–metabolome–epigenome interactions may help to generate novel information about pathways linking alterations in early microbial colonization to later adiposity. This study protocol proposes a comprehensive, longitudinal, multi-omics approach in a mother–infant cohort. The recruitment within the third trimester of pregnancy and the subsequent follow-up of the offspring will allow us to explore both prenatal and postnatal exposures. Moreover, the evaluation of maternal profiles offers the opportunity to identify transgenerational patterns that could provide a potent tool to generate prevention strategies against obesity development even before birth, as well as to understand how maternal metabolic, microbial, and epigenetic status influences offspring metabolic state and related health trajectories.

Summarizing, this study will generate novel insights into the early-life determinants of adiposity by combining epigenetic, metagenomic, and metabolomic data. The identification of predictive biomarkers may enable earlier and more effective prevention strategies, ultimately reducing the burden of obesity and related metabolic diseases later in life.

## Figures and Tables

**Table 1:** Schedule of assessments and follow-up visits.

Study Period							
	Enrollment	Follow-Up					Close-Out
	t_1_	t_2_	t_3_	t_4_	t_5_	t_6_	t_7_
Enrollment:							
Eligibility screen	X						
Informed consent	X						
Inclusion criteria	X						
Exclusion criteria	X						
Assessments:							
Clinical Assessment	X						
Anthropometric measurements (mother)	X						
Food frequency questionnaires (FFQ)	X						
Quality of life questionnaires	X						
Ultrasound	X						
Buccal swab (mother)	X	X					
Blood (mother)	X	X					
Stool (mother)	X	X					
Buccal swab (infant)		X	X	X	X	X	X
Stool (Infant)		X	X	X	X	X	X
Anthropometric measurements (infant)		X	X	X	X	X	X

t_1_: 38 weeks of gestation; t_2_: 0–72 h postpartum; t_3_: 1 month; t_4_: 6 months; t_5_: 1 year; t_6_: 2 years; t_7_: 3 years. “X” indicates that the corresponding procedure is performed at that study time point.

## Supplementary Materials

The following supporting information can be downloaded at: https://www.mdpi.com/article/10.3390/jcm14196694/s1, Supplementary Material S1: Information Sheet.

## Abbreviations

The following abbreviations are used in this manuscript:

HCUVA	Virgen de la Arrixaca University Hospital
SCFA	Short-chain fatty acids
BCAA	Branched-chain amino acids
FFQ	Food frequency questionnaires
