# Supplementary material for: Dynamics of the Epigenome, Microbiome, and Metabolome in Relation to Early Adiposity in the Maternal–Infant Axis: Protocol for a Prospective, Observational Pilot Study in the Spanish NEMO Cohort
Source: J Clin Med. 2025 Sep 23;14(19):6694. doi: 10.3390/jcm14196694 (PMC12525363; doi:10.3390/jcm14196694)
Supplement: Supplementary file 1 [file jcm-14-06694-s001.zip › jcm-3801008-supplementary.pdf]

HOJA DE INFORMACIÓN PARA PARTICIPANTES EN ESTUDIOS DE INVESTIGACIÓN CON MUESTRAS

Título del estudio: "Evaluación de la dinámica del epigenoma, microbioma y metaboloma y su interacción con el aumento de la adiposidad en el eje materno-infantil".
Investigadores Principales, servicio/unidad y centro: María Ángeles Núñez Sánchez, Instituto Murciano de Investigación Biosanitaria (IMIB-Arrixaca); José Eliseo Blanco Carnero, Hospital Clínico Universitario Virgen de la Arrixaca (HCUVA); Manuel Sánchez-Solís de Querol, Hospital Clínico Universitario Virgen de la Arrixaca (HCUVA).
Nº de versión y fecha: VERSIÓN 3 (29/05/2024)

INTRODUCCIÓN:

Considerando el estado de gestación en el que se encuentra, nos dirigimos a usted para informarle sobre un estudio en el que se le invita a participar. Nuestra intención es que reciba la información correcta y suficiente para que pueda evaluar y juzgar si quiere o no participar en este estudio. Antes de decidir si quiere participar o no, le rogamos lea detenidamente este documento que incluye la información sobre este proyecto. Puede formular todas las preguntas que le surjan y solicitar cualquier aclaración sobre cualquier aspecto del mismo. Nosotros le aclararemos las dudas que puedan surgir en cualquier momento. Además, puede consultar con las personas que considere oportuno.

El proyecto cuenta con el informe favorable de un Comité de Ética de la Investigación acreditado en España.

PARTICIPACIÓN VOLUNTARIA:

Debe saber que su participación es voluntaria y que puede decidir no participar o cambiar su decisión y retirar el consentimiento en cualquier momento, sin que por ello se altere la relación con su médico ni se produzca perjuicio alguno en su tratamiento.

PROPÓSITO Y PROCEDIMIENTOS DEL ESTUDIO:

En la actualidad, la obesidad infantil está alcanzando niveles epidémicos y es una causa de preocupación en el mundo desarrollado. Dado que los niños obesos tienen más probabilidad de ser obesos en la edad adulta y desarrollar enfermedades metabólicas asociadas como diabetes tipo 2, hipertensión o enfermedad del hígado graso no alcohólica, se necesitan urgentemente estrategias que permitan actuar de manera preventiva. Es por ello por lo que este proyecto pretende identificar biomarcadores no invasivos asociados al aumento de la adiposidad en los primeros meses de vida, así como la relación que existe entre dichos biomarcadores en la madre y los presentes en el hijo. Por tanto, en este proyecto se empleará un enfoque multidisciplinar para evaluar en profundidad los factores de riesgo de padecer obesidad en etapas tempranas de la vida. Dadas las connotaciones éticas asociadas a la obtención de muestras biológicas como, por ejemplo, sangre, en edades tempranas, los resultados obtenidos en este proyecto pueden suponer un alto impacto en la práctica clínica. Además, la identificación de nuevos biomarcadores no invasivos implicados en el aumento de la adiposidad tiene un gran impacto socioeconómico ya que permitirá desarrollar estrategias de prevención personalizadas desde edades tempranas disminuyendo así el coste asociado al desarrollo de comorbilidades.

PROCEDIMIENTOS DE OBTENCIÓN DE MUESTRAS, MOLESTIAS Y POSIBLES RIESGOS:

Algunas de las muestras se obtienen durante el seguimiento habitual de su enfermedad o proceso; otras son solicitadas porque son necesarias para cumplir con los objetivos de este estudio. A continuación, le explicamos cuáles son y los riesgos asociados a los procedimientos utilizados para su obtención.

Todas las voluntarias embarazadas que cumplan los criterios de inclusión y acepten participar en el estudio aportarán una muestra de heces, orina, raspado bucal y análisis de sangre en el tercer trimestre de gestación (en torno a la semana 38 de gestación) y el día del parto, así como muestras de leche materna (cuando sea posible) 24-48 h tras el parto, al mes y a los seis meses posparto. Así mismo, se obtendrán muestras de sangre del cordón umbilical y placenta el día del parto, y muestras de heces y raspado bucal el primer día de vida, al mes, a los seis meses, al año, a los dos años y a los 3 años de vida del infante previa autorización por parte de ambos progenitores (cuando proceda). El proyecto cumplirá en todo momento los principios fundamentales establecidos en la Declaración de Helsinki.

A su vez, se realizará una evaluación clínica completa de las voluntarias gestantes, que incluirá el registro de datos antropométricos, información sobre antecedentes personales o médicos (los hábitos tóxicos o el consumo crónico de medicamentos son criterios de exclusión), y evaluación de la ingesta nutricional utilizando cuestionarios de frecuencia alimentaria (FFQ) y de la calidad de vida. También se realizará una evaluación del crecimiento y metabolismo fetal en el último trimestre de gestación, así como una exploración física del neonato para la obtención de datos antropométricos y el tipo de nutrición del niño.

Las muestras y los datos asociados se mantendrán bajo las condiciones de seguridad adecuadas y se garantiza que los sujetos no podrán ser identificados a través de medios considerados razonables por personas distintas a las autorizadas.

Es posible que sea necesario algún dato o muestras adicionales. En ese caso, su médico se pondrá en contacto con usted para solicitarle de nuevo su colaboración. Se le informará de los motivos y se le solicitará de nuevo su consentimiento (ver opción sí/no al final de la hoja).

Si ha decidido participar en el estudio, deberá:

- Facilitar que se le realice una historia clínica, se le tomen muestras de raspado bucal, y sangre, así como suministrar muestras de heces y orina.
- Facilitar que se tomen muestras de sangre del cordón umbilical y placenta inmediatamente después del parto.
- Suministrar muestras de leche en distintos tiempos después del parto, heces del neonato, y facilitar que se le tomen muestras de raspado bucal.

BENEFICIOS ESPERADOS:

No se espera un beneficio directo por su participación en el estudio. No obstante, los conocimientos obtenidos gracias a los estudios llevados a cabo a partir de sus muestras y de muchas otras pueden ayudar al avance médico y, por ello, a otras personas. No percibirá ningún beneficio económico por la donación de las muestras y la cesión de los datos proporcionados, ni tendrá derechos sobre posibles beneficios comerciales de los descubrimientos que puedan conseguirse como resultado de la investigación efectuada.

LUGAR DE ANÁLISIS Y ALMACENAMIENTO DE LAS MUESTRAS:

Durante el desarrollo del estudio sus muestras pueden ser analizadas en diversos laboratorios y se mantendrán almacenadas durante 5 años, en previsión de que fuera necesario repetir algún análisis adicional relacionado con los objetivos del estudio. Durante este proceso el responsable de las muestras será el investigador/promotor del proyecto.

INFORMACIÓN SOBRE EL DESTINO DE LAS MUESTRAS Y USO FUTURO

Una vez finalizado el estudio, las muestras sobrantes serán destruidas, a no ser que usted consienta para que puedan ser almacenadas y utilizadas en futuras investigaciones. La finalidad del almacenamiento de estas muestras es que sean utilizadas en proyectos de investigación en el futuro.

Tanto el almacenamiento durante la realización de la investigación como destino de las muestras al término de la misma será el Biobanco del IMIB-Arrixaca (perteneciente al Registro Nacional de Biobancos del Instituto de Salud Carlos III con número de referencia PT17/0015/0038). Desde allí se cederán para proyectos autorizados, posiblemente también en el extranjero, previo dictamen favorable del comité científico y del comité de Ética del Biobanco. Usted podrá dirigirse al biobanco para obtener información de los proyectos en los que se hayan utilizado sus muestras. El paciente deberá firmar el consentimiento general de donación al Biobanco del IMIB-Arrixaca. Los datos que se deriven de la utilización de estas muestras en futuras investigaciones se tratarán del mismo modo que el resto de datos que se obtengan durante este estudio (ver apartado de confidencialidad).

DERECHO DE REVOCACIÓN DEL CONSENTIMIENTO:

Si cambiara de opinión en relación con la donación de las muestras biológicas y la cesión de los datos proporcionados, tiene derecho a solicitar su destrucción o anonimización, a través de su médico/investigador/investigador principal de la colección/ biobanco. No obstante, debe saber que los datos que se hayan obtenido en los análisis realizados hasta ese momento podrán ser utilizados para los fines solicitados y podrán conservarse en cumplimiento de las obligaciones legales correspondientes.

CONFIDENCIALIDAD/PROTECCIÓN DE DATOS PERSONALES:

Sus muestras estarán asociadas a un código (codificadas) Sólo personal autorizado (personal con perfil biosanitario asociado al proyecto) podrá relacionar la información derivada de los análisis realizados con información sobre su identidad.

A este estudio les son plenamente de aplicación la Ley Orgánica 3/2018 de 5 de diciembre, de Protección de datos de carácter Personal y garantía de los derechos digitales y el Reglamento (UE) 2016/679 del Parlamento europeo y del Consejo de 27 de abril de 2016 de Protección de Datos (RGPD). Por ello, es importante que conozca la siguiente información:

- Sus datos personales serán tratados con la finalidad indicada en el documento objeto de firma y serán conservados durante los años necesarios para cumplir con la normativa vigente aplicable.

- El Responsable del Tratamiento es el Hospital Clínico Universitario "Virgen de la Arrixaca" (Área I de Salud-Murcia/Oeste), cuyo **Delegada de Protección de Datos** (DPD) es Doña **Elena García Quiñones**

con dirección en Servicio Murciano de Salud, C./ Central nº 7, Edificio Habitamia I, 30100, Espinardo-Murcia (correo electrónico: **dpd-sms@carm.es**).

- La base jurídica que legitima el tratamiento es su consentimiento

- Normativa aplicable: *Reglamento (UE) n 536/2014 del Parlamento Europeo y del Consejo, de 16 de abril de 2014 , sobre los ensayos clínicos de medicamentos de uso humano, y por el que se deroga la Directiva 2001/20/CE; Ley Orgánica 3/2018, de 5 de diciembre, de Protección de Datos de Carácter Personal y Garantía de los Derechos Digitales, Ley 14/2007, de 3 de julio, de Investigación biomédica; Real Decreto Legislativo 1/2015, de 24 de julio, por el que se aprueba el texto refundido de la Ley de garantías y uso racional de los medicamentos y productos sanitarios; Ley 44/ Ley 44/2003, de 21 de noviembre, de ordenación de las profesiones sanitarias, así como la Ley 14/1986, de 25 de abril, General de Sanidad, la Ley 41/2002, de 14 de noviembre, de autonomía del paciente, y demás legislación vigente en materia sanitaria.*

- Sus datos no serán cedidos, salvo en los casos obligados por Ley o en casos de urgencia médica. No obstante, en todo momento podrá revocar el consentimiento prestado, así como ejercer sus derechos de **acceso, rectificación, supresión, oposición, limitación del tratamiento y portabilidad,** en la medida que sean aplicables, a través de comunicación escrita al Responsable del Tratamiento de la siguiente manera **concretando su solicitud, junto con su DNI o documento equivalente**:

    - **Investigadores Principales del estudio**: Dr. José Eliseo Blanco Carnero, Dr. Manuel Sánchez-Solís de Querol, Dra. María Ángeles Núñez Sánchez.
    - **Domicilio**: Hospital Clínico Universitario "Virgen de la Arrixaca", Ctra. Murcia-Cartagena s/n, CP 30120, El Palmar-Murcia.

- Asimismo, le informamos de la posibilidad de presentar una reclamación ante la Agencia Española de Protección de Datos (C/Jorge Juan, 6 Madrid 28001) www.agpd.es

El acceso a su información personal quedará restringido al médico del estudio/colaboradores, Autoridades Sanitarias en materia de inspección, al Comité Ético de Investigación Clínica, cuando lo precisen para comprobar los datos y procedimientos del estudio, pero siempre manteniendo la confidencialidad de los mismos.

Los datos recogidos para el estudio estarán identificados mediante un código, de manera que no se incluya información que pueda identificarle, y sólo su médico del estudio/colaboradores podrá relacionar dichos datos con usted y con su historia clínica.

A partir de dichos datos se podrán elaborar comunicaciones científicas para ser presentadas a congresos o revistas científicas siempre manteniendo en todo momento la confidencialidad de sus datos de carácter personal.

Se le informa que de conformidad a lo previsto en la Disposición adicional decimoséptima de la Ley Orgánica 3/2018, de 5 de diciembre, de Protección de Datos de Carácter Personal y garantía de los derechos digitales, así como del artículo 89 del Reglamento (UE) 2016/679, en el caso que con sus datos se lleve a cabo un tratamiento con fines de investigación en salud pública y, en particular, biomédica se procederá a:

- Realizar una evaluación de impacto que determine los riesgos derivados del tratamiento en los supuestos previstos en el artículo 35 del Reglamento (UE) 2016/679 o en los establecidos por la

autoridad de control. Esta evaluación incluirá de modo específico los riesgos de reidentificación vinculados a la anonimización o seudonimización de los datos.

- Someter la investigación científica a las normas de calidad y, en su caso, a las directrices internacionales sobre buena práctica clínica.

- Adoptar, en su caso, medidas dirigidas a garantizar que los investigadores no acceden a datos de identificación de los interesados. En el supuesto de que no pueda garantizarse esta separación entre los datos y el investigador, se le garantiza un compromiso expreso de confidencialidad por parte del investigador, así como de no realizar ninguna actividad de reidentificación. Se adoptarán medidas de seguridad específicas para evitar la reidentificación y el acceso de terceros no autorizados.

IMPLICACIONES DE LA INFORMACIÓN OBTENIDA AL ANALIZAR LAS MUESTRAS:

En el caso de que usted lo solicite, se le podrá facilitar información acerca de los estudios de investigación en los que se hayan utilizado sus muestras, así como de los resultados generales del presente estudio.

En el caso de que en este estudio se obtengan datos que pudieran ser clínica o genéticamente relevantes para usted, e interesar a su salud o a la de su familia, podrá solicitar que le sean comunicados por su médico del ensayo si así lo indica en la casilla que aparece al final de este documento. No obstante, si el paciente hubiera indicado su negativa y cuando esta información, según criterio del médico responsable, sea necesaria para evitar un grave perjuicio para su salud o la de sus familiares biológicos, se informará a un familiar próximo o a un representante, previa consulta al Comité de Ética Asistencial del centro. La comunicación de esta información se llevará a cabo por profesionales que le podrán explicar adecuadamente su relevancia y las opciones que se pudieran plantear. En caso de información genética clínicamente relevante podrá recibir el preceptivo consejo genético.

INVESTIGACIONES FUTURAS:

Autorizo la posible reutilización de datos personales con fines de investigación en materia de salud y biomédica para finalidades o áreas de investigación relacionadas con este estudio.

HOJA DE CONSENTIMIENTO INFORMADO

Título del proyecto: "Evaluación de la dinámica del epigenoma, microbioma y metaboloma y su interacción con el aumento de la adiposidad en el eje materno-infantil".

Investigadores Principales, servicio/unidad y centro: José Eliseo Blanco Carnero, Hospital Clínico Universitario Virgen de la Arrixaca (HCUVA); Manuel Sánchez-Solís de Querol, Hospital Clínico Universitario Virgen de la Arrixaca (HCUVA); María Ángeles Núñez Sánchez, Instituto Murciano de Investigación Biosanitaria (IMIB-Arrixaca).

Datos del participante/paciente
Nombre

____

Investigador o persona que proporciona la información
Nombre

____

1. He leído, he sido informado y comprendo el contenido de la presente hoja de Información, lo que acredito con mi firma en prueba de mi consentimiento en todo lo que en ella se contiene.

2. Entiendo que mi participación es voluntaria y gratuita y comprendo que puedo solicitar la revocación de este consentimiento en cualquier momento, sin tener que ofrecer explicaciones y sin que esto repercuta en mis cuidados médicos presentes y/o futuros.

1. Deseo que el médico del estudio me comunique la información derivada de la investigación que pueda ser relevante y aplicable para mi salud o la de mis familiares:

   ▪ SI ▪ NO Teléfono o e-mail de contacto.........................

2. Consiento al almacenamiento y uso de las muestras y de los datos asociados para futuras investigaciones en las condiciones explicadas en esta hoja de información.

   ▪ SI ▪ NO

3. Consiento a ser contactado en el caso de necesitar más información o muestras biológicas adicionales.

   ▪ SI ▪ NO Teléfono o e-mail de contacto.........................

Fecha: Firma del Participante/paciente

Fecha: Firma del Investigador o persona que proporciona la información

HOJA DE CONSENTIMIENTO INFORMADO

Título del proyecto: "Evaluación de la dinámica del epigenoma, microbioma y metaboloma y su interacción con el aumento de la adiposidad en el eje materno-infantil".

Investigadores Principales, servicio/unidad y centro: José Eliseo Blanco Carnero, Hospital Clínico Universitario Virgen de la Arrixaca (HCUVA); Manuel Sánchez-Solís de Querol, Hospital Clínico Universitario Virgen de la Arrixaca (HCUVA); María Ángeles Núñez Sánchez, Instituto Murciano de Investigación Biosanitaria (IMIB-Arrixaca).

Datos del progenitor o progenitores (cuando proceda):
Nombre/s

____

Investigador o persona que proporciona la información
Nombre

____

3. He leído, he sido informado y comprendo el contenido de la presente hoja de Información, lo que acredito con mi firma en prueba de mi consentimiento en todo lo que en ella se contiene.

4. Entiendo que mi participación es voluntaria y gratuita y comprendo que puedo solicitar la revocación de este consentimiento en cualquier momento, sin tener que ofrecer explicaciones y sin que esto repercuta en mis cuidados médicos presentes y/o futuros.

4. Deseo que el médico del estudio me comunique la información derivada de la investigación que pueda ser relevante y aplicable para mi salud o la de mis familiares:

   ▪ SI ▪ NO Teléfono o e-mail de contacto.........................

5. Consiento al almacenamiento y uso de las muestras y de los datos asociados para futuras investigaciones en las condiciones explicadas en esta hoja de información.

   ▪ SI ▪ NO

6. Consiento a ser contactado en el caso de necesitar más información o muestras biológicas adicionales.

   ▪ SI ▪ NO Teléfono o e-mail de contacto.........................

Fecha: Firma del/los progenitores

Fecha: Firma del Investigador o persona que proporciona la información

REVOCACION DEL CONSENTIMIENTO

Yo, D/Dña... revoco el consentimiento prestado en fecha y no deseo continuar participando en el estudio "Evaluación de la dinámica del epigenoma, microbioma y metaboloma y su interacción con el aumento de la adiposidad en el eje materno-infantil".

Firma del paciente:

Firma del investigador: Fecha: Fecha
